# Reconstruction of Diverse Verrucomicrobial Genomes from Metagenome Datasets of Freshwater Reservoirs

**DOI:** 10.3389/fmicb.2017.02131

**Published:** 2017-11-02

**Authors:** Pedro J. Cabello-Yeves, Rohit Ghai, Maliheh Mehrshad, Antonio Picazo, Antonio Camacho, Francisco Rodriguez-Valera

**Affiliations:** ^1^Evolutionary Genomics Group, Departamento de Producción Vegetal y Microbiología, Universidad Miguel Hernández, San Juan de Alicante, Spain; ^2^Department of Aquatic Microbial Ecology, Institute of Hydrobiology, Biology Centre of the Academy of Sciences of the Czech Republic, České Budějovice, Czechia; ^3^Cavanilles Institute of Biodiversity and Evolutionary Biology, University of Valencia, Valencia, Spain

**Keywords:** freshwater *Verrucomicrobia*, metagenomics, rhodopsin, nitrogen fixation, genome streamlining

## Abstract

The phylum *Verrucomicrobia* contains freshwater representatives which remain poorly studied at the genomic, taxonomic, and ecological levels. In this work we present eighteen new reconstructed verrucomicrobial genomes from two freshwater reservoirs located close to each other (Tous and Amadorio, Spain). These metagenome-assembled genomes (MAGs) display a remarkable taxonomic diversity inside the phylum and comprise wide ranges of estimated genome sizes (from 1.8 to 6 Mb). Among all *Verrucomicrobia* studied we found some of the smallest genomes of the Spartobacteria and Opitutae classes described so far. Some of the Opitutae family MAGs were small, cosmopolitan, with a general heterotrophic metabolism with preference for carbohydrates, and capable of xylan, chitin, or cellulose degradation. Besides, we assembled large copiotroph genomes, which contain a higher number of transporters, polysaccharide degrading pathways and in general more strategies for the uptake of nutrients and carbohydrate-based metabolic pathways in comparison with the representatives with the smaller genomes. The diverse genomes revealed interesting features like green-light absorbing rhodopsins and a complete set of genes involved in nitrogen fixation. The large diversity in genome sizes and physiological properties emphasize the diversity of this clade in freshwaters enlarging even further the already broad eco-physiological range of these microbes.

## Introduction

Freshwater *Verrucomicrobia* were originally classified by a largely morphology-based taxonomic approach into a broad taxon called the “Caulobacterales,” a diverse group of microbes bearing “prosthecae” (Henrici and Johnson, 1935). These prosthecate microbes were later understood to belong to multiple bacterial phyla, e.g., *Proteobacteria* and *Verrucomicrobia*. Several phylogenetic analyses, first based on 16S rRNA sequences from cultured isolates and environmental clone libraries, and expanded later using phylogenomic methods grouped them together with two other bacterial phyla, the *Planctomycetes* and *Chlamydiae*, together comprising what is now referred to as the PVC superphylum (Wagner and Horn, 2006; Gupta et al., 2012). More recently, the phyla *Lentisphaerae* and OP3 have been added to this superphylum. While the presence of “wart-like structures,” i.e., *verrucae*, was identified as a distinguishing feature of the model organism, *Verrucomicrobium spinosum*, several members of *Verrucomicrobia* share a much more intriguing compartmentalized cellular organization with the *Planctomycetes* and also some *Chlamydiae*, suggesting a common ancestry for the group at large (Lee et al., 2009). The nature and function for these compartmentalized structures in the PVC superphylum remains as yet unclear, but hypotheses proposing homologous relationships to eukaryotic intracellular structures (Devos and Reynaud, 2010; Forterre and Gribaldo, 2010; Lonhienne et al., 2010; Santarella-Mellwig et al., 2010) that were advanced have been refuted (McInerney et al., 2011). On the other hand, in anaerobic ammonium oxidizing *Planctomycetes* the function of the organelle anammoxosome is well studied (Van Teeseling et al., 2013).

*Verrucomicrobia* have been detected in diverse habitats. Several species have been isolated from soil (Schlesner, 1987; Sangwan et al., 2004; Kant et al., 2011a), and culture-independent studies have estimated that *Verrucomicrobia* may account for up to 20% of total bacterial communities in soil (Bergmann et al., 2011). Verrucomicrobial isolates display a wide variety of features, e.g., the methanotrophs *Methylacidiphilum* and *Methylacidimicrobium* from volcanic environments (Dunfield et al., 2007; Pol et al., 2007; Islam et al., 2008; van Teeseling et al., 2014) able to perform nitrogen fixation (Hou et al., 2008; Khadem et al., 2010, 2012b; Erikstad and Birkeland, 2015), aerobes or anaerobes from rice paddy soils (Chin et al., 2001; Kant et al., 2011b). A considerable number have been isolated in association to animal guts, including termites (Isanapong et al., 2012; Wertz et al., 2012) and cephalotes (Lin et al., 2016), while mucin degraders have been found in the human microbiome (Derrien et al., 2004). Some isolates have been identified associated to fruits (grape parasites) and plants (Okazaki et al., 2014; Brown et al., 2015). Representatives from aquatic environments have been described in recent years, for instance: marine isolates associated to sponges, corals, and algae (Scheuermayer et al., 2006; Yoon et al., 2007, 2008).

Several ecological studies have detected *Verrucomicrobia* in suboxic ponds (Briée et al., 2007), tropical lakes (De Wever et al., 2008), wetlands (Zhang et al., 2014), and associated to cyanobacterial blooms (Eiler and Bertilsson, 2004). Fluorescent *in situ* hybridization (FISH) experiments targeting specific *Verrucomicrobia* clades in humic lakes have shown that freshwater *Verrucomicrobia* are diverse and may have large populations up to ca. 19% of the total community (Arnds et al., 2010). Highly variable abundances (6–17%) have also been reported in shotgun metagenomic studies in freshwater lakes/reservoirs (Lonhienne et al., 2010; Oh et al., 2011; Eiler et al., 2014; Ghai et al., 2014).

However, very few genomes of *Verrucomicrobia* have been described from the aquatic habitat, i.e., water column, but some single cell-amplified genomes (SAGs) inferred to be polysaccharide-degraders have been sequenced from the marine environment (Martinez-Garcia et al., 2012a) and two metagenome-assembled genomes (MAGs) (Tamagnini et al., 2002), one from the brackish environment of the Baltic Sea (Herlemann et al., 2013) and the other from a water treatment plant (*Verrucomicrobia* bacterium Ga0077533) are available. Only a single SAG has been described from the freshwater Lake Mendota (Martinez-Garcia et al., 2012a). Even though there is a growing number of *Verrucomicrobia* isolates, MAGs and SAGs in the databases, the genomic information from pelagic freshwater habitats is scarce and limited largely to 16S rRNA surveys. Therefore, the ecological role of these unusual microbes in freshwaters remains obscure.

Here we have used metagenomic tools to reconstruct novel freshwater *Verrucomicrobia* from two reservoirs (Amadorio and Tous) located in Southeast Spain. The large number of verrucomicrobial genomes (18) reconstructed from these freshwater reservoirs show broad differences in genomic make up within the same verrucomicrobial class Opitutae, likely reflecting specialization in different ecological strategies (oligotrophic K-strategists versus copiotrophic r-strategists). The data presented contribute a large number of novel verrucomicrobial groups detected for the first time through these MAGs.

## Materials and Methods

### Sampling, Physicochemical Profiles, Sequencing, Assembling, and Annotation

A total of five datasets from two different Spanish freshwater reservoirs were used in this work. Four of them have already appeared in publications and came from winter season, when the water column was mixed. Two from Amadorio reservoir (Ghai et al., 2014) and another two from Tous reservoir (Cabello-Yeves et al., 2017). In this work we describe for the first time another metagenomic sample that was taken from Tous reservoir on July 21, 2016 at 13 m depth, when the water column was stratified. Following the same assembly and annotation methods previously described in Amadorio and Tous winter metagenomics studies (Ghai et al., 2014; Cabello-Yeves et al., 2017), sequencing of the 0.22 μm filter was performed using Illumina HiSeq 4000 (Oklahoma Medical Research Foundation, United States). A total of 250 million sequence reads (paired end 2 × 150 bp) were produced for the 13 m summer sample from Tous reservoir. The metagenome was assembled using the IDBA-UD assembler (Peng et al., 2012) with mink 70, maxk 100, step 10, and pre-correction parameters, obtaining a total of 4892 contigs larger than 10 Kb. Gene predictions on the assembled contigs were assessed using Prodigal in metagenomic mode (Hyatt et al., 2010), tRNAs were predicted using tRNAscan-SE (Lowe and Eddy, 1997), and ribosomal rRNA genes were identified using ssu-align (Nawrocki and Eddy, 2010) and meta-rna (Huang et al., 2009). Comparisons of predicted protein sequences against NCBI NR, COG (Tatusov et al., 2001), and TIGFRAM (Haft et al., 2001) databases were performed for taxonomic binning and functional annotation.

### Unassembled 16S rRNA Verrucomicrobial Classification in Different Freshwater Datasets

In order to estimate the percent of verrucomicrobial 16S rRNA reads in different metagenomics datasets we firstly prepared a non-redundant version of the RDP database by clustering all available 16S rRNA coding sequences (approximately 2.3 million) into approximately 800,000 sequences at 90% identity level using UCLUST (Edgar, 2010). This database was used to identify candidate 16S rRNA reads in the Illumina datasets (unassembled). If a read matched this database at an *e*-value < 1*e*-5 it was considered a potential 16S rRNA gene fragment. These candidate reads were aligned to archaeal, bacterial, and eukaryal 16S/18S rRNA HMM models using ssu-align to identify true 16S/18S sequences (Nawrocki and Eddy, 2010). The final sequences were compared to the entire RDP database and classified into a high level taxon if the sequence identity was ≥80% and the alignment length was ≥90 bp. Sequences failing these thresholds were discarded.

### Identification of Verrucomicrobial Contigs and *de Novo* Genomic Reconstruction

*Verrucomicrobia* MAGs from Amadorio were reconstructed using the small and large fractions of Amadorio reservoir datasets (Ghai et al., 2014), while genomes from Tous winter (February) metagenomes were reconstructed using the assembly from 12 and 25 m samples (Cabello-Yeves et al., 2017). *Verrucomicrobia* genomes from Tous summer sample were reconstructed with a single sample from 13 m, as described above. Contigs longer than 10 kb were considered verrucomicrobial when hits (BLASTP) to this phylum were obtained for >60% of the total genes presented in each contig. More specifically, contigs falling within these parameters were binned using taxonomy, principal component analysis of tetranucleotide frequencies, GC content, and coverage values in Tous and Amadorio metagenomes (Ghai et al., 2014; Cabello-Yeves et al., 2017). Tetranucleotide frequencies were computed using compseq program in the EMBOSS package (Rice et al., 2000). Principal component analysis was performed using the FactoMineR package in R (Lê et al., 2008). To estimate the genome size and completeness of the assembled genomes we used the CheckM package with the parameters taxonomy_wf and specific *Verrucomicrobia* marker set of genes (Parks et al., 2015). Average Nucleotide Identity (ANI) among our MAGs was also calculated (Goris et al., 2007).

### Phylogenomic Trees

The phylogenomic placement of the newly described verrucomicrobial MAGs was assessed with PhyloPhlAn tool (Segata et al., 2013). The complete genomes of representatives from all known *Verrucomicrobia* classes and reconstructed MAGs of this study were added to the built-in tree of life in PhyloPhlAN. PhyloPhlAN uses USEARCH (Edgar, 2010) to identify the conserved proteins and subsequent alignments against the built-in database are performed using MUSCLE (Edgar, 2004). Finally, an approximate maximum-likelihood tree is generated using FastTree (Price et al., 2010) with local support values using Shimodaira–Hasegawa test (Shimodaira and Hasegawa, 1999). We used 58 reference *Verrucomicrobia* genomes (MAGs, SAGs, and isolates) with >50% of genome completeness assessed by CheckM (Parks et al., 2015) from the different existing subdivisions and classes inside the phylum.

### Single Gene Trees

A 16S rRNA tree was made with the *Verrucomicrobia* and *Planctomycetes* 16S rRNA references and those sequences retrieved from Amadorio and Tous metagenomes, either from binned or unbinned *Verrucomicrobia* MAGs. Sequences were aligned using MAFFT (Katoh et al., 2002), which uses the four-way consistency objective function for incorporating structural information. Trees were constructed with FastTree2 (Price et al., 2010), using a GTR model, a gamma approximation, and 100 bootstraps. We did not observe major differences between MAFFT and MUSCLE alignments for 16S phylogenies. The same method was applied for the NifHDK protein concatenate tree, which was constructed with NifHDK concatenate from a wide range of bacteria (Boyd, 2000; Boyd et al., 2011, 2015; Boyd and Peters, 2013; McGlynn et al., 2013). A batch of 200 rhodopsins from multiple origins were aligned using MUSCLE (Edgar, 2004) as previously described in other freshwater rhodopsin phylogenies (Ghai et al., 2014). A maximum-likelihood tree was constructed with FastTree2 (Price et al., 2010), JTT+CAT model, a gamma approximation, and 100 bootstraps.

### Metagenomic Read Recruitment

Metagenome reads mapping to our MAGs were performed against more than 150 publicly available freshwater and brackish metagenomes using BLASTN with at least 50 bp of length and >95% identity and an *e*-value of ≤1*e*-5. These hits were used to compute the abundance expressed as RPKG (reads recruited per kilobyte of genome per gigabyte of metagenome). RPKG is a normalized number comparable across metagenomes from different locations. All metagenomic datasets used in this work are publicly available: Amadorio (Ghai et al., 2014) and Tous reservoirs (Cabello-Yeves et al., 2017), Lake Lanier (Oh et al., 2011), Dexter reservoir (PRJNA312985), Lake Baikal (PRJNA396997, SRR5896114, and SRR5896115), Klamath Iron Gate Dam (PRJNA312830), Lake Houston (PRJNA312986), Amazon Lakes (Toyama et al., 2016), Lake Mendota (PRJNA330170, PRJNA330171, and PRJNA330042), Swedish Lakes, and Trout Bog (Eiler et al., 2014).

### Metabolic Analysis

The metabolic features of each genome were analyzed with RAST annotation pipeline database (Overbeek et al., 2013), BlastKoala with phylum-specific genes (Kanehisa et al., 2016), Batch Web CD-Search Tool with default parameters (Marchler-Bauer et al., 2016), and BLASTP, BLASTN, and TBLASTX (Altschul et al., 1997). We annotated each MAG protein set with the dbCAN (Yin et al., 2012) and CAZY enzyme databases (Lombard et al., 2013).

### Microscopy and FISH

For microscopic counts of heterotrophic bacterioplankton, water samples were fixed *in situ* with a paraformaldehyde: glutaraldehyde solution to a final concentration in the sample of 1%: 0.05% (w/v) (Marie et al., 1997). Once in the laboratory, subsamples (5–10 mL) were filtered through 0.2-μm-pore-size black filters (Nuclepore, Whatman). For quantification, a quarter of a filter was stained with 4,6-diamidino-2-phenylindole (DAPI) (Porter and Feig, 1980) (SIGMA) and counted with an inverted Zeiss III RS epifluorescence microscope (1250×, resolution 0.02857 lm/pixel) using a G365 exciting filter, LP420 suppression filter for blue light, and G546 exciting filter, LP590 suppression filter for green light (MacIsaac and Stockner, 1993). Water samples fixed with paraformaldehyde to 2% final concentration then filtered within the next 2 h through white polycarbonate filters (0.2 μm pore size). Sections were stained with both the different oligonucleotide probes and DAPI and subsequently mounted for microscopic evaluation. The previously described probe EUB338-III (GCTGCCACCCGTAGGTGT) was used to detect the phylum *Verrucomicrobia* (Daims et al., 1999). FISH-specific probes for the *Verrucomicrobia* taxa from Amadorio and Tous Reservoirs were also designed using the PRIMER3 tool (Rozen and Skaletsky, 1999) from specific sequences obtained from the metagenomes. Specifically, the 16s rRNA probe VrmC9LTo (ATAGGCCGCGGGCTCCTCAA) was designed to detect the specific Verrucomicrobia-Tous-C9LFEB from Tous Reservoir and the rRNA probe VrmG3Ama (AGGCCGCAAGCTCCTCCTGA) to detect Opitutae-AMD-G3. All probes were labeled with the indocarbocyanine dye Cy3 in 5′ (Thermo Fisher Scientific, Waltham, MA, United States). Probes matching sequences were checked for specificity using the Probe Match RDP (Cole et al., 2009), RDP Release 11, Update 5: September 30, 2016 (3,356,809 16S rRNAs database^1^). The FISH protocol was performed as previously described (Sekar et al., 2003). Hybridization conditions for VrmC9LTo and VrmG3Ama were adjusted by formamide (VWR BDH Prolabo) series applied to different subsamples, with a final optimal formamide concentration of 35% in both cases. Optimal concentration of formamide used for EUB338-III was 40%. Relative densities of hybridized bacteria were calculated as the product of their relative abundances on filter sections (percentage of DAPI-stained objects) and the DAPI-stained direct cell counts. The absolute abundance values were revised based on the values obtained in independent DAPI counts without the hybridization process. A minimum of 500 probe-stained cells were counted per sample. Total percent of heterotrophs was also determined independently. DAPI-stained objects were counted in order to confirm that the total percent did not vary with the hybridized cells. Images from FISH determinations were analyzed using the NIH ImageJ Software to determine cell dimensions^2^.

## Results and Discussion

### Verrucomicrobial 16S rRNA Abundance in Freshwater Habitats

We have estimated the relative abundance of verrucomicrobial 16S rRNA in several available freshwater metagenomic datasets, ranging from oligotrophic to eutrophic and tropical to cold, distributed in different latitudes and altitudes. We have found a relative abundance between 2 and 19% (Supplementary Figure 1), much higher than some values estimated for marine water column and sediments (2 and 1.4%, respectively) (Freitas et al., 2012). In Lake Baikal, Lake Lanier, and Dexter reservoir the abundance of *Verrucomicrobia* was particularly high, reaching 20% of the total 16S rRNA reads of these datasets. No correlation of verrucomicrobial abundances was apparent either with latitude or with trophic status, although it is evident that they are found in all kinds of freshwater lakes or reservoirs.

### Reconstruction of Novel Verrucomicrobial Genomes

In order to simplify the nomenclature of the work presented here, all reconstructed *Verrucomicrobia* will be referred to as MAGs which come from an assorted clonal population from a single species. Based on the metagenomics sequencing, assembly, annotation, and binning procedures described before (see section “Materials and Methods”) we were able to reconstruct a total of 18 *Verrucomicrobia* genomes (**Table 1**) of which 15 were different genomes as suggested by ANI calculations. Only three pairs of genomes displayed an ANI higher than 98% suggesting they came from the same microbe (Supplementary Figure 2). A 100% ANI between Opitutae-Tous-C4FEB and Opitutae-Tous-C5TDCM was found, which suggested that the same microbe was obtained from two different samples (summer and winter) from the same reservoir. Along the same lines, another genome (Verrucomicrobiae-Tous-C3TDCM and Verrucomicrobiae-AMD-G2 with 100% ANI between them) was assembled twice from the two different reservoirs, Tous and Amadorio that are about 100 km apart. Finally, Opitutae-AMD-G3 and Opitutae-Tous-C2FEB shared 98% ANI values (same species). Estimations of genome sizes ranged from 1.83 to 6.18 Mb and eight genomes appeared to be more than 80% complete (**Table 1**). Some of these genomes appear to be the smallest verrucomicrobial representatives described yet (Supplementary Figure 3A), with a wide variation in GC content (51–70%), with the smaller genomes generally having a lower GC content (Supplementary Figure 3B).

**Table 1:** Summary statistics for the 18 *Verrucomicrobia* metagenome-assembled genomes (MAGs) from Tous and Amadorio reservoirs.

*Verrucomicrobia*	Number	Sequence	GC	Median	Coding	Completeness	Contamination	Estimated	Origin	Class
MAG	of	length		intergenic	density			genome		
	CDS	(Mbp)		spacer (bp)				size (Mb)		
**Opitutae-AMD-G1**	1479	1.54	64.07	38	90	67.84	0.14	2.27	Amadorio	Opitutae
**Opitutae-AMD-G3**	1720	1.90	64.52	37	94	85.58	3.74	2.14	Amadorio	Opitutae
**Opitutae-Tous-C10FEB**	1333	1.42	56.80	58	93	57.89	0.41	2.44	Tous	Opitutae
Opitutae-Tous-C1TDCM	3363	4.15	67.69	74	92	74.9	1.02	5.48	Tous	Opitutae
**Opitutae-Tous-C2FEB**	1236	1.33	64.70	35	94	68.16	2.86	1.89	Tous	Opitutae
Opitutae-Tous-C4FEB	3144	3.76	62.76	66	91	94.95	0.82	3.93	Tous	Opitutae
Opitutae-Tous-C5TDCM	3223	3.99	62.94	68	91	82.65	5.16	4.58	Tous	Opitutae
**Opitutae-Tous-C8FEB**	1810	2.17	69.98	34	95	67.76	0	3.21	Tous	Opitutae
Pedosphaera-Tous-C6FEB	4618	5.69	64.58	76	91	87.85	35.78	4.16	Tous	Pedosphaera
**Spartobacteria-AMD-G4**	1782	1.82	56.62	35	92	80.27	0.5	2.25	Amadorio	Spartobacteria
**Spartobacteria-AMD-G5**	617	0.56	58.59	18	94	29.59	0	1.89	Amadorio	Spartobacteria
**Spartobacteria-Tous-C9RFEB**	900	0.92	54.14	33	94	50.16	0	1.83	Tous	Spartobacteria
Verrucomicrobiae-AMD-G2	2798	3.26	52.06	63	91	96.08	0.82	3.36	Amadorio	Verrucomicrobiae
Verrucomicrobiae-Tous-C2TDCM	1612	1.85	61.72	63	93	55.07	0.41	3.34	Tous	Verrucomicrobiae
Verrucomicrobiae-Tous-C3TDCM	2628	2.96	52.00	62	91	87.62	0.41	3.37	Tous	Verrucomicrobiae
Verrucomicrobiae-Tous-C4TDCM	3690	4.28	63.94	69	91	66.98	3.27	6.18	Tous	Verrucomicrobiae
**Verrucomicrobiae-Tous-C5FEB**	1751	1.77	57.92	43	94	55.8	9.07	2.89	Tous	Verrucomicrobiae
Verrucomicrobia-Tous-C9LFEB	3585	4.28	57.29	68	90	90.61	2.04	4.63	Tous	Unclassified

*Completeness, contamination, and estimated genome size (Mb) parameters were assessed by CheckM. Genomes considered small (<3.2 Mb and <60 bp of median intergenic spacer) are highlighted in bold*.

**FIGURE 1:**
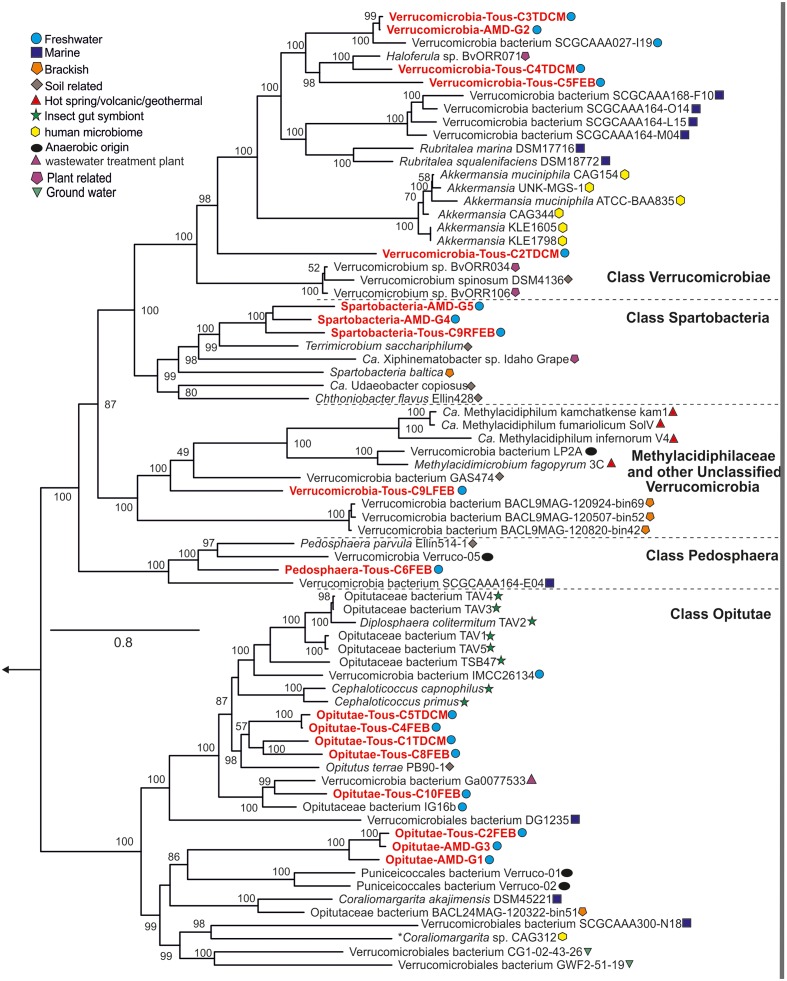
Phylogenomics of the entire phylum *Verrucomicrobia*. Reference isolates, MAGs, and SAGs from multiple habitats and classes (Opitutae, Verrucomicrobiae, Spartobacteria, Pedosphaera, and Unclassified) and subdivisions are represented in the protein concatenated tree. Tous and Amadorio MAGs presented in this study are red colored.

Currently, three classes are defined within the phylum *Verrucomicrobia*: Opitutae (subdivision 4), Spartobacteria (subdivision 2), and Verrucomicrobiae (subdivision 1). There are also two subdivisions (6 and 7) which comprise unclassified representatives and another one comprising *Pedosphaera parvula* like microbes (subdivision 3). Recently, one divergent member from the verrucomicrobial subdivision 5 has been described as a novel phylum-level lineage (Spring et al., 2016). A complete phylogenomic tree with the reference verrucomicrobial genomes and the 18 MAGs assembled in this work is shown in **Figure 1**. A 16S rRNA phylogenetic tree with reference verrucomicrobial sequences and those sequences retrieved from MAGs is also provided (Supplementary Figure 4). Among the 18 novel *Verrucomicrobia*, we reconstructed eight genomes belonging to the class Opitutae, five to the class Verrucomicrobiae, three to class Spartobacteria, one to class Pedosphaera, and one related to the unclassified *Verrucomicrobia*, which contain representatives of the Methylacidiphilaceae family (Op den Camp et al., 2009). It is quite remarkable that these recovered genomes appear in widely divergent taxonomic units attesting to the broad diversity of verrucomicrobial representatives inhabiting freshwater environments.

**FIGURE 2:**
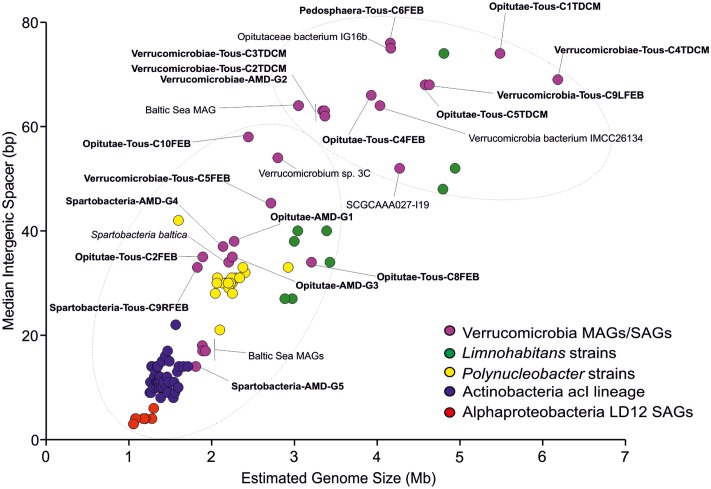
Estimated genome size (Mb) versus median intergenic spacer (bp) scatter plot of Tous and Amadorio *Verrucomicrobia* MAGs compared to other freshwater microbes. We include all known brackish and freshwater *Verrucomicrobia*, representatives of the streamlined freshwater AcI lineage *Actinobacteria* (Ghai et al., 2014; Kang et al., 2017; Neuenschwander et al., 2017), freshwater *Alphaproteobacteria* LD12 SAGs (Zaremba-Niedzwiedzka et al., 2013), freshwater heterotrophic *Limnohabitans* strains and members of the cosmopolitan and abundant *Polynucleobacter* genus. Estimated genome sizes have been calculated based on checkM completenesses for each MAG, SAG, strain, or cultured genome. Tous and Amadorio MAGs are highlighted in bold.

The genome sizes estimated from these MAGs suggest we have obtained two types of microbes (**Figure 2**), firstly small verrucomicrobial genomes, with relatively smaller intergenic spacers compared to those seen in reference marine (*Coraliomargarita akajimensiis* or *Rubritalea marina*), freshwater (*Opitutaceae bacterium* IG16b, *Verrucomicrobia bacterium* SCGCAAA027-I19), or brackish genomes (*Spartobacteria baltica*). These small genomes belong to the class Opitutae within the family Puniceicoccales (Opitutae-AMD-G1, Opitutae-AMD-G3, and Opitutae-Tous-C2FEB) and the class Spartobacteria (Spartobacteria-AMD-G4, Spartobacteria-AMD-G5, and Spartobacteria-Tous-C9RFEB). They have estimated genome sizes of 1.83–2.27 Mb, with median intergenic spacers smaller than 38 bp (Supplementary Figure 3A). These Spartobacteria and Opitutae–Puniceicoccae genomes described here are among the smallest *Verrucomicrobia* genomes discovered so far. Secondly, we have reconstructed large *Verrucomicrobia* genomes, larger than 3.5 Mb (Verrucomicrobia-Tous-C9LFEB or Opitutae-Tous-C4FEB), reaching in some cases 5 Mb (Pedosphaera-Tous-C6FEB), which exhibit larger genome sizes, although still smaller than the 6–8 Mb seen in soil representatives (e.g., *V. spinosum*), symbionts of termites (*Diplosphaera colitermitum* or *O. bacterium* TAV1, TAV3, TAV4, or TAV5) or associated to sugar beet plants (*Haloferula BVOrr071*). Only a single genome was estimated to have a size >6 Mb (Verrucomicrobiae-Tous-C4TDCM, 6.18 Mb).

The verrucomicrobial genomes have been plotted by estimated genome sizes and median intergenic spacers (as indicators of how streamlined the genomes are) together with other typical freshwater microbes that go from the very streamlined K-strategists (acI *Actinobacteria* or LD12 *Alphaproteobacteria*) to relatively large genomes of r-strategists like *Limnohabitans* (**Figure 2**). It is evident how the genomes presented in this work cover the whole range of streamlining from one end to the other. Overall, we have obtained a great diversity of genomes from all known *Verrucomicrobia* classes, although we were able to reconstruct more genomes belonging to the class Opitutae. We have focused our efforts in a comparative study between the two kinds of Opitutae genomes, that appear to correspond also to a separation between endemic and cosmopolitan microbes (see below).

### Abundant versus Rare *Verrucomicrobia*

In this study we have retrieved MAGs coming from summer stratification and winter mixing periods from Tous reservoir. Specifically, four of the Tous DCM (deep chlorophyll maximum) reconstructed *Verrucomicrobia* were found exclusively associated to the DCM of the stratified summer sample (Supplementary Figure 5). This seems to indicate that the DCM has a specific verrucomicrobial population associated to it. However, most MAGs were present at much higher abundances at the Tous 25 m metagenome. One likely explanation, being largely heterotrophic microbes specialized in the degradation of polymers (see below), is that they accumulate at the deepest part of the water body where particulate material sinking in the water column would be more abundant. A different distribution pattern was found for the photoheterotrophic Opitutae-Tous-C4FEB (and the nearly identical Opitutae-Tous-C5TDCM) and Verrucomicrobiae-Tous-C5FEB, which showed very similar recruitment values in the three depths tested for Tous, either 12 and 25 (winter sample) or 13 m (summer stratified sample). One of these, Opitutae-Tous-C4FEB, was also found at similar abundances in Amadorio 10 m metagenome (winter sample). Both Opitutae-Tous-C4FEB and Verrucomicrobiae-Tous-C5FEB contain proteorhodopsin genes (see below) and photoheterotrophic potential which could help extend their range to more superficial waters.

Abundances of all MAGs were tested by mapping the reads of different freshwater datasets to our MAGs (Supplementary Figure 6). In general, we observed that small Opitutae genomes were more abundant in all tested metagenomes compared to the rest of the MAGs retrieved from different classes. The small Opitutae-Puniceicoccae (Opitutae-AMD-G1, Opitutae-AMD-G3, and Opitutae-Tous-C2FEB) representatives mainly recruit in Tous 25 m metagenome, in contrast with the low abundances found in Tous 12 m metagenome, which suggests that these microbes are likely associated to the deeper parts of Tous reservoir. Interestingly, we also observed high abundances for Opitutae-AMD-G1 and Opitutae-AMD-G3/Opitutae-Tous-C2FEB in Lake Baikal, the ultraoligotrophic largest freshwater body on Earth (Cabello-Yeves et al., submitted for publication) (suffering from 5-month frozen cycles) compared to the more temperate Spanish reservoirs. They were also found at lower abundances in Klamath Iron Gate Dam and lakes Mendota, Erken, and Lanier suggesting a good adaption to a wide range of lakes, ranging from oligotrophic (Tous reservoir) to eutrophic (Lake Mendota) and temperate (Tous or Amadorio) to cold (Lake Baikal or Swedish lakes). These data suggest that these microbes could be a very abundant subclass of Opitutae members in many different freshwater bodies, as confirmed by the recruitment plots (Supplementary Figures 7, 8). We also detected values of 10 RPKG (1× coverage) for Opitutae-AMD-G1/G3 in samples associated to cyanoblooms from Dexter reservoir, showing evidence of an association between (pico) cyanobacteria and *Verrucomicrobia* (Eiler and Bertilsson, 2004). Abundance was also assessed in the same datasets for some reference freshwater *Verrucomicrobia*, finding low RPKG values for all of them (Supplementary Figure 5).

**FIGURE 3:**
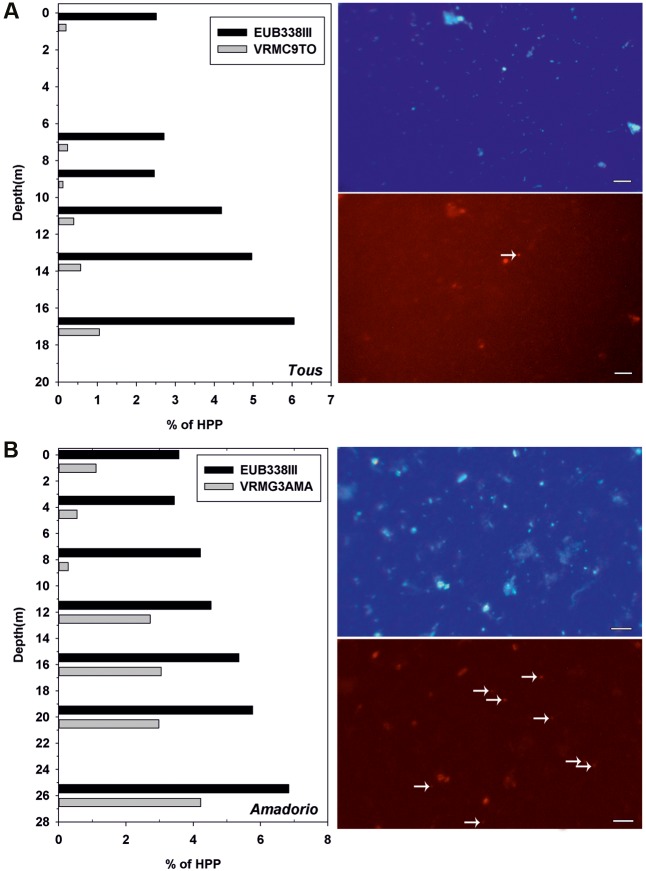
(Left), relative abundance (%) of total *Verrucomicrobia* (Eub338III) and specific Verrucomicrobia-Tous-C9LFEB (VrmC9to, 4.63 Mb of estimated genome size) and Opitutae-AMD-G3 (VrmG3Ama, 2.13 Mb of estimated genome size) relative to total heterotrophic picoplankton (HPP) for **(A)** Tous and **(B)** Amadorio reservoirs. (Right), microscopic fluorescence *in situ* hybridization images of samples from **(A)** Tous reservoir and **(B)** Amadorio reservoir. The micrographs show two pairs of identical microscopic fields, with samples stained with DAPI (up) and with the specific designed probes labeled with Cy3 (down). White arrows mark VrmC9To **(A)** and VrnG3Ama **(B)**. Bar: 5 μm (all four pictures).

The distribution of total planktonic *Verrucomicrobia* (EUBB-III) and the specific groups (Verrucomicrobia-Tous-C9LFEB for Tous and Opitutae-AMD-G3 for Amadorio) in the vertical profile (samples were taken every 2 m depth and fixed for microscopy) under summer stratification conditions was addressed by FISH in both reservoirs (**Figure 3**). The distribution pattern in the vertical profile of the percentage of total *Verrucomicrobia* with respect to the total heterotrophs was similar in both reservoirs, with their relative contribution increasing with depth. In both cases the lowest values were observed in the upper waters (epilimnion) with an average of 2.5% in Tous and a 2.8% in Amadorio, gradually increasing toward the bottom of the reservoirs with deep maxima of *Verrucomicrobia* contribution to total bacterioplankton of ca. 6% in Tous and 7% in Amadorio. FISH counts using the VrmC9LTo-specific probe of Verrucomicrobia-Tous-C9LFEB show that this group represents only a small part of the total heterotrophs of Tous Reservoir during summer stratification, always below 1%, with a relatively constant contribution of ca. 15% of total *Verrucomicrobia* within the water column. The VrmG3Ama probe, which is specific of Opitutae-AMD-G3 from Amadorio reservoir, allowed us determining that this group was present at a relatively low contribution in relation to total heterotrophs (<1%), however, from 12 m depth to the bottom its abundance increased to 3–4% with respect to the total of heterotrophs and contributed over 50% of the total *Verrucomicrobia*. These data confirm that Opitutae-AMD-G3 (2.13 Mb of estimated genome size) is a very abundant microbe in Amadorio (and also in Tous as was previously assessed by recruitment), which fits with the proposal that these microbes are K-strategists, while Verrucomicrobia-Tous-C9LFEB (large genome of 4.63 Mb of estimated genome size) contributed at very low numbers in Tous (as was also tested by recruitment), which fits with the idea that it possesses an opportunistic strategy.

### Carbohydrate-Based Metabolism in Freshwater Verrucomicrobia

In general, we found an aerobic heterotrophic metabolism with degradation of polysaccharides in all of our genomes, containing metabolic pathways involved in glycolate, glucarate/galactorate, glucoronate/galacturonate, gluconate, polysaccharides, monosaccharides, and aminosugars utilization and degradation. We also noticed a large amount of Ton/Tol/Exb receptors for transport of polymers, vitamins, and iron uptake, and carbon storage preference for glycogen (which is both synthesized and degraded) instead of poly-[(beta)]-hydroxybutyrate (PHB). Interestingly, *Verrucomicrobia* appear to be involved in the metabolism of two glycosphingolipids, galactosylceramides, and sulfatides, a pathway that is typically found in eukaryotes, presenting several enzymes like aryl-sulfatases, sialidases, and beta-galactosidases. Among the diverse microbes reconstructed in this work, these features were widespread and common to the majority of them, although certain pathways for mono and polysaccharide utilization were specific to some (see below). Despite the general aerobic and heterotrophic metabolism inferred, we were able to identify some microbes with anaerobic reductase complexes (Pedosphaera-Tous-C6FEB), some of them could be able to perform photoheterotrophic lifestyles through rhodopsin pumps and one of them could be capable of nitrogen fixation.

In addition to more simple sugars, *Verrucomicrobia* are known to be able to tackle more complex and recalcitrant polysaccharides (Martinez-Garcia et al., 2012a). We performed a protein annotation for each MAG in order to explore the presence of glycosyl hydrolases (GHs) and other polysaccharide degrading enzymes based on the CAZY (Lombard et al., 2013) and dbCAN (Yin et al., 2012) databases. An overview of the GH families presented in each MAG is shown in the Supplementary Data Sheet 1. In general, we observed that the reconstructed freshwater *Verrucomicrobia* are fundamentally chitin degraders as they chiefly contain GH109 (α-*N*-acetylgalactosaminidase). We also detected some other abundant GHs families as GH33 (sialidase or neuraminidase), GH74 (endoglucanase and xyloglucanase) and GH2 (β-galactosidase, β-mannosidase, or β-glucuronidase), GH13_9 (α-amylase), and GH77 (amylomaltase or 4-α-glucanotransferase). In general, we detected a growing proportion of GHs with the increase in MAG genome size and the total number of CDS. It also correlates with a higher number of transporters and metabolic pathways found in the larger genomes compared to the small genomes (see below). It is noteworthy that Verrucomicrobia-Tous-C9LFEB contained the highest number of GHs (218), a huge number compared to the other MAGs, which contained between 2 and 80 GHs. This microbe, as explained below, contains the highest and most remarkable variety of metabolic pathways of all the genomes compared here, including nitrogen fixation and efficient degradation of recalcitrant compounds such as chitin, cellulose, xylan, starch, or mannan, among others.

### Metabolic Overview of Small versus Large Verrucomicrobial Genomes

**FIGURE 4:**
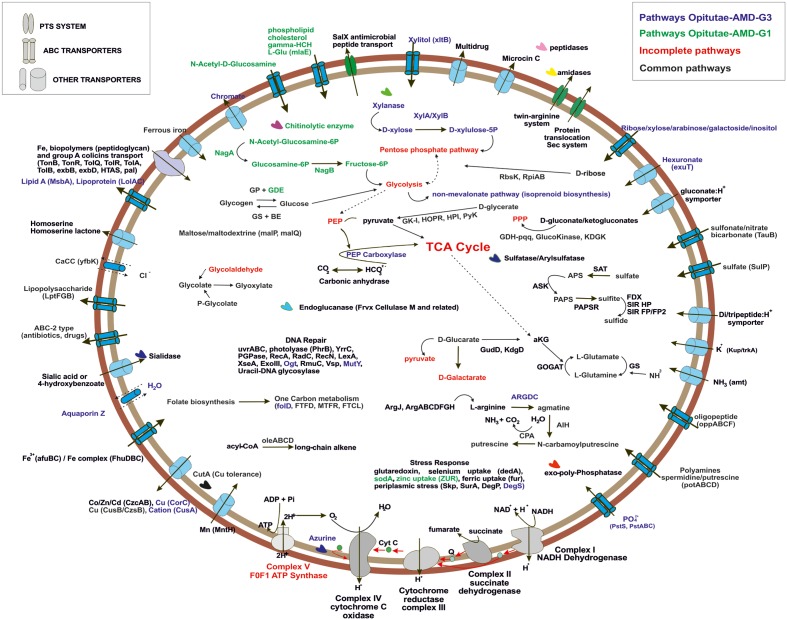
Metabolic overview of Opitutae-AMD-G1 and Opitutae-AMD-G3 *Verrucomicrobia* genomes. Pathways are color coded. Common pathways are colored in black; incomplete pathways are red colored; specific pathways to Opitutae-AMD-G1 are green; and specific pathways to Opitutae-AMD-G3 are dark blue.

**FIGURE 5:**
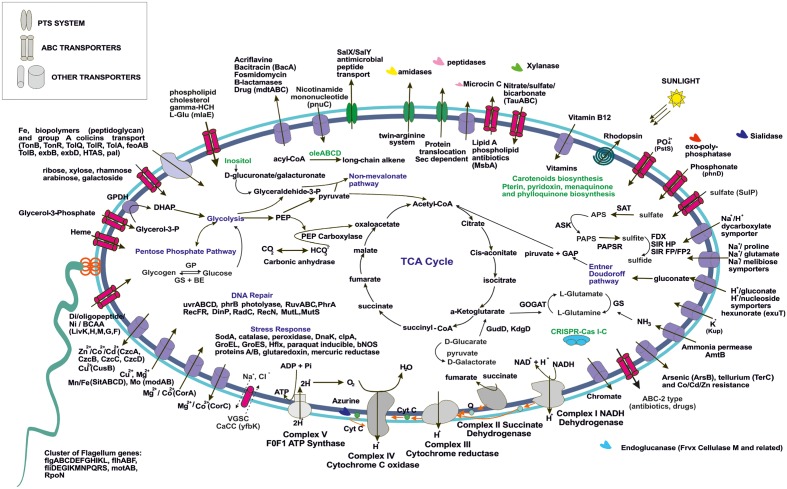
Metabolic overview of Opitutae-Tous-C4FEB genome. All pathways shown are complete in the genome.

To simplify the work presented here, we have analyzed in depth the metabolic pathways found in four of our MAGs. We focused on the two small and cosmopolitan Opitutae-AMD-G1 and Opitutae-AMD-G3 (**Figure 4**), which have genome size estimations of around 2.2 Mb, being among the smallest *Verrucomicrobia* representatives ever found, at least in freshwater ecosystems. We also studied more in depth two of the largest genomes that appear sporadically in metagenomes (see above), Opitutae-Tous-C4FEB (**Figure 5**) and Verrucomicrobia-Tous-C9LFEB (Supplementary Figure 9), which have estimated sizes of 3.9 and 4.6 Mb, respectively, and contain a rhodopsin and nitrogen fixation, respectively.

#### C-metabolism

The ability of these microbes to use glycogen instead of PBH as carbon storage source is interesting, and has also been reported in acidophilic nitrogen fixing methanotrophs (Khadem et al., 2010, 2012a). Genes for glycogen biosynthesis (*glgA*, *glgB*, *glgC*, *glgP*) were common to both small and large genomes, although glycogen debranching enzyme (glgX) was only detected in the small Opitutae-AMD-G1. A specific operon (*oleABCD*) that has been previously described for long chain alkene biosynthesis from acyl-CoA in some members of *Planctomycetes*, *Verrucomicrobia*, *Actinobacteria*, *Chloroflexi*, and *Betaproteobacteria* (Sukovich et al., 2010), was only found in these Opitutae genomes, either large or small, and appears to be a specific carbon storage of this class.

One of the most remarkable features of these freshwater *Verrucomicrobia* (independently of their genome size) was their ability to effectively degrade different types of mono and polysaccharides (as described above). Sialidases (including one sialic acid transporter), which release *N*-acetylneuraminate residues from glycans, glycoproteins, and polysaccharides (Moncla et al., 1990; Kim et al., 2011) were found in the four genomes. Endoglucanases (Frvx), coding for cellulase M, were inferred from both large and small genomes. Degradation of xylan, a major group of hemicellulose present in plant cell walls and some algae, was detected in the big genomes and the small Opitutae-AMD-G3 but not in the small Opitutae-AMD-G1. Conversely, we found a specific pathway for *N*-acetyl-D-glucosamine (chitin) degradation in small Opitutae-AMD-G1 and big Opitutae-Tous-C4FEB (although only one chitinolytic enzyme was found in the small Opitutae-AMD-G1). Complete pathways with GHs for mannose and rhamnose utilization were only detected in the unclassified Verrucomicrobia-Tous-C9LFEB. The two large genomes were able to utilize sugar alcohol carbon sources, since they have a glycerol-3-phosphate transporter and a glycerol-3-phosphate dehydrogenase. Pathways for inositol catabolism to form glucuronate and transformation of the latter to glyceraldehyde-3-phosphate, as well as fructose utilization via PTS transport system were also common in these large genomes.

#### Transporters

Generally, we detected a high number of Exb, Ton, and Tol biopolymer and iron transporters in all the reconstructed genomes, although we also noticed an increase in number of these transporter with increasing genome size. The presence of these transporters suggests an alternative route for incorporation and recycling of dissolved organic matter in freshwater bodies, which was already described in marine ecosystems (Tang et al., 2012). In general we could identify a higher number of transporters for heavy metals (some of them conferring resistance to arsenite and tellurium), branched chain amino acids, di-oligo-peptides, heme, or multidrug resistance in the larger genomes.

Both large and small genomes contain exo-polyphosphatases, inorganic pyrophosphatases, phosphate starvation proteins (PhoH), alkaline phosphatase, or inorganic phosphate transporters, which suggest that *Verrucomicrobia*, like freshwater *Actinobacteria*, are important in inorganic phosphorous sequestering which could contribute to prevent eutrophication (Ghai et al., 2014). The storage of polyphosphate was also assessed in the methanotroph *Ca*. Methylacidiphilum fumariolicum SolV (Khadem et al., 2012a). Polyamines have been described in some members of the PVC superphylum in freshwater systems, especially in *Planctomycetes* (Griepenburg et al., 1999); only small genomes presented a spermidine/putrescine transporter (potABCD) together with a cluster of putrescine biosynthesis from L-arginine and agmatine.

#### Other Ecologically Relevant Genes

Bacterial nitric oxide synthases have been described in the past years as enzymes involved in toxin biosynthesis, protection against oxidative stress, and regulation of recovery from radiation damage (Correa-Aragunde et al., 2013). In Opitutae-Tous-C4FEB genome one gene encoding a nitric oxide synthase was detected; the presence of this gene was also common to other reconstructed MAGs. However, we did not detect any potential toxin-related genes adjacent to this gene as has been described previously in Streptomyces (Kers et al., 2004).

We found a cluster of flagellum genes for motility functions exclusively in the large genomes (Opitutae-Tous-C4FEB and Verrucomicrobia-Tous-C9LFEB), which was totally absent in the rest of the reconstructed genomes of this work. A CRISPR-Cas type I-C was also found in Opitutae-Tous-C4FEB. Some of the main differences between small and large genomes were based on their ability to perform other different types of metabolism which couple with the general heterotrophic metabolism in *Verrucomicrobia*. For instance, Opitutae-Tous-C4 (**Figure 5**), a 3.9 Mb microbe (estimated genome size) contains almost all of the pathways described in the small Opitutae (see above) plus other pathways suggesting a photoheterotrophic metabolism, including a complete pathway for carotenoids biosynthesis together with a proteorhodopsin proton-pump. Carbonic anhydrases, phosphoenolpyruvate carboxylase, or PhrB photolyases for stress response to light were also detected. Compared to the small genomes, we found more vitamin (pterines, pyridoxine, menaquinone, and phylloquinone) and cofactor biosynthetic pathways, including one vitamin B12 transporter in Opitutae-Tous-C4.

### Nitrogen Fixation in Freshwater *Verrucomicrobia*

Although *Verrucomicrobia*, including methanotrophs and termite symbionts (Khadem et al., 2010; Wertz et al., 2012), have been previously reported to be capable of fixing nitrogen, we are describing for the first time the possible ability of a truly freshwater *Verrucomicrobia* (Verrucomicrobia-Tous-C9LFEB) for nitrogen fixation. Typically, a minimum set of six *nif* genes is required in bacteria for nitrogen fixation, three of them code for structural and catalytic components (NifHDK) and the other three for biosynthetic proteins (NifENB) (Dos Santos et al., 2012). We found a complete *nif* operon containing the six *nifHDKENB* subunits.

**FIGURE 6:**
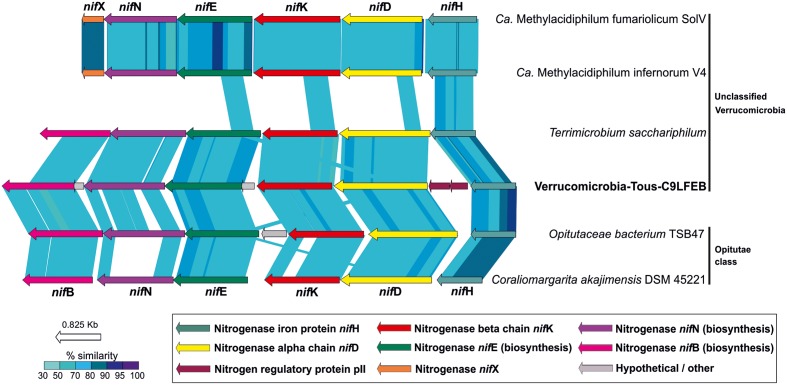
Structure and synteny of the nitrogenase operon among different *Verrucomicrobia*. A minimum set of six proteins are represented in the operon. NifH, NifD, and NifK comprise the catalytic domain of the nitrogenase. NifN, NifE, and NifB comprise the biosynthetic domain. Comparison made with TBLASTX with >50% similarity hits and 50 bp alignment lengths.

A phylogeny of the NifHDK concatenate among different molybdenum, vanadium, and iron nitrogenases from different bacterial phyla representatives (Boyd et al., 2011, 2015; Boyd and Peters, 2013; McGlynn et al., 2013) (Supplementary Figure 10) suggests that the nitrogenase described here affiliates with a cluster of molybdenum *Verrucomicrobia* nitrogenases which have representatives in termite guts (*O. bacterium* TAV5 and TSB47), soil (*Terrimicrobium sacchariphilum*), and sea (*C. akajimensiis*). Similar to what can be seen for the entire operon (**Figure 6**) these nitrogenases are more divergent compared to the verrucomicrobial *Methylacidiphilum* representatives (Khadem et al., 2010).

A comparison of the *nif* operon found in Verrucomicrobia-Tous-C9LFEB with some representatives of the *Verrucomicrobia* phylum is shown in **Figure 6**. The *nif* operon is largely syntenic across these microbes, with the exception of an additional *nifX* gene in the *Methylacidiphilum* representatives. However, Verrucomicrobia-Tous-C9LFEB *nif* cluster is more similar to the one found before in Opitutae, even though the genome itself is phylogenetically closer to unclassified *Verrucomicrobia* (**Figure 1**). Another peculiar feature of the operon is the apparent insertion/translocation of the gene encoding the pII nitrogen regulatory protein, between *nifD* and *nifH* genes. Moreover, the pII gene also appears to be duplicated.

Nitrogenase activity is commonly limited to anaerobic environments, where nitrogenase is not inhibited by oxygen, although some aerobic microorganisms like *Cyanobacteria* (Esteves-Ferreira et al., 2017) or *Azotobacter vinelandii* (Bishop et al., 1980) present strategies to perform nitrogen fixation under aerobic conditions. The discovery of the nitrogen fixing Verrucomicrobia-Tous-C9LFEB is remarkable since the study site (Tous reservoir) is totally oxic throughout the year. However, the anaerobic nitrogen fixing activity may be facilitated by anoxic microenvironments in cell aggregates or particles regardless of the oxygen content of the water column (Dang and Lovell, 2016). This microbe also contains nitrate and nitrite ammonification pathways, which could be used as an alternative pathway for ammonia production, especially when nitrogen fixation leads to high energetic expenses for the cell.

### Novel Verrucomicrobial Rhodopsins

**FIGURE 7:**
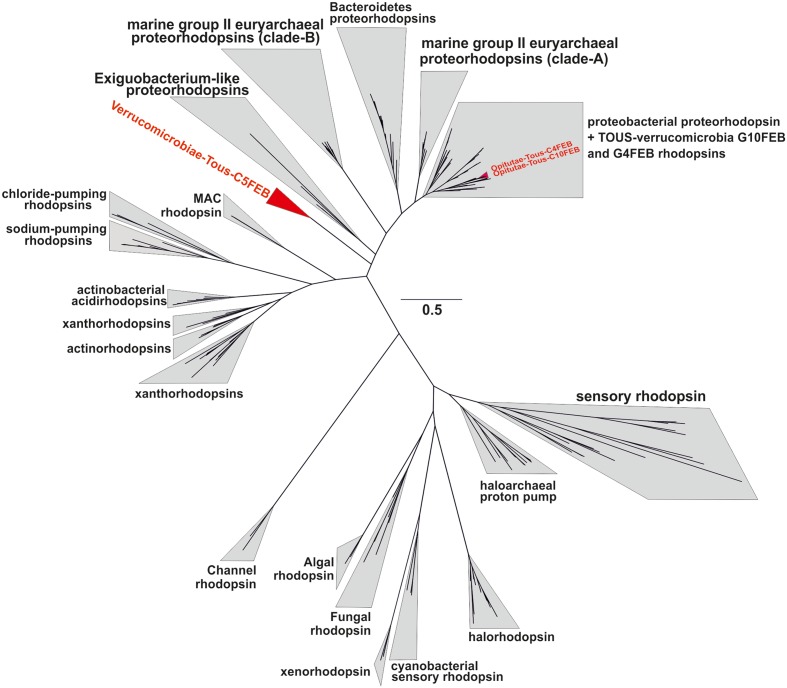
Phylogenetic placement of the three Tous freshwater verrucomicrobial rhodopsins together with a batch of 200 rhodopsin references from different types and multiple origin (marine, brackish, freshwater, or halophilic).

It has been suggested that the *Verrucomicrobia* phylum could have representatives with photoheterotrophic metabolism via rhodopsin pumps (Martinez-Garcia et al., 2012b). In this manuscript we report three novel rhodopsins, the ones from the Opitutae-Tous-C4FEB and Opitutae-Tous-C10FEB genomes, clearly belong to the marine proteorhodopsin clade (**Figure 7**). Additionally, Verrucomicrobiae-Tous-C5FEB contains one novel rhodopsin belonging to a new clade of rhodopsin proton pumps, its closest sequences being the rhodopsins from *Exiguobacterium* (Gushchin et al., 2013). This rhodopsin could be part of a novel branch characteristic of *Planctomycetes–Verrucomicrobia* PVC superphylum representatives. The key residues of these three rhodopsins, compared to other representatives (Supplementary Figure 11) and based on the presence of the L-105 or M-105 residues in the retinal pocket, clearly show that the ones described here are green light absorbing proton pumps (Man et al., 2003). Actually, it is remarkable that all rhodopsins from Tous metagenomes were green-light variants.

## Conclusion

*Verrucomicrobia* is a relatively novel phylum which has acquired interest in the scientific community, mainly because of their global distribution in different types of environments and hosts. Analyzing metagenomes from freshwater reservoirs in Spain we could reconstruct a remarkable diversity of relatively complete verrucomicrobial-assembled genomes. They covered a broad range of genome sizes and streamlining levels (judging from the mean intergenic spacers). This great size variability of reconstructed genomes, ranging from about 2 to 6 Mb, would shed light onto the classification inside the phylum. We found a correlation between both ends of the genome size spectrum and the distribution in diverse freshwater and brackish metagenomes around the world with some small genomes being extremely widespread and abundant and others, particularly the large genomes, with much more restricted distribution. Although all the genomes seem to correspond to aerobic heterotrophs degrading polysaccharides, the larger genomes display much more transport potential and have likely more diversified metabolism. There is a group that appears to be adapted to the stratified period at DCM depths while the majority of genomes appear to be more abundant in deeper waters (Supplementary Figure 5), which are richer in sinking particles that could be the ecological niche of many of these microbes. The uneven distribution of larger genomes could just reflect a blooming strategy that makes their presence sporadic while the smaller genomes are probably more constant in their presence being less demanding and likely also slower growing (K-strategists).

We found a nitrogen fixing cluster, a characteristic never described before in free living freshwater *Verrucomicrobia* and (excluding cyanobacteria) not typical from freshwater well-oxygenated systems like Tous reservoir, which never undergoes anoxic conditions. The photoheterotrophic metabolism in some of the microbes presented here was confirmed with the discovery of freshwater *Planctomycetes–Verrucomicrobia* rhodopsins, likely a novel branch within this already hyperdiversified family of proteins. It is noteworthy that the small Opitutae-AMD-G3 and Opitutae-AMD-G1 described here are cosmopolitan in lakes from different environmental conditions, ranging from cold oligotrophic lakes (Lake Baikal or Swedish Lakes), temperate oligotrophic (Lake Lanier or Tous reservoir), and temperate mesotrophic (Amadorio) or eutrophic (Lake Mendota). We have visualized by FISH the microbes represented by Opitutae-AMD-G3 and Verrucomicrobia-Tous-C9LFEB and would like to propose *Candidatus* names for both microbes: *Candidatus* Opitutus lacustris AMD-G3 and *Candidatus* Verrucococcus diazotrophicus Tous-C9LFEB.

## Data Accessibility

Tous reservoir metagenomic datasets have been deposited in the NCBI SRA database with BioProject number PRJNA342151 (Tous February 12 m: SRR4198666; Tous February 25 m: SRR4198832; Tous July 13 m: SRR5338504). Amadorio reservoir metagenomic datasets are available under BioProject number PRJNA238866. The 18 assembled *Verrucomicrobia* genomes have been deposited in the NCBI under Biosample identifiers SAMN06820742-SAMN06820759.

## Author Contributions

RG, FR-V, and PC-Y conceived this work. PC-Y and AP performed the sample collection and filtration. PC-Y performed the DNA extraction. Analysis was carried out by PC-Y, RG, MM, and AP. Manuscript was written by PC-Y, FR-V, and RG. All authors read and approved the final manuscript.

## Conflict of Interest Statement

The authors declare that the research was conducted in the absence of any commercial or financial relationships that could be construed as a potential conflict of interest.

## Footnotes

1 https://rdp.cme.msu.edu/index.jsp

2 http://rsb.info.nih.gov/ij/index.html
