# Supplementary material for: Reconstruction of Diverse Verrucomicrobial Genomes from Metagenome Datasets of Freshwater Reservoirs
Source: Front Microbiol. 2017 Nov 2;8:2131. doi: 10.3389/fmicb.2017.02131 (PMC5673642; doi:10.3389/fmicb.2017.02131)
Supplement: Supplementary file 1 [file Presentation_1.PDF]

Supplementary Material

Reconstruction of diverse verrucomicrobial genomes from metagenome datasets of freshwater reservoirs

PEDRO J. CABELLO-YEVES[1], ROHIT GHAI[2], MALIHEH MEHRSHAD[2], ANTONIO PICAZO[3], ANTONIO CAMACHO[3] and FRANCISCO RODRIGUEZ-VALERA[1*]

[1]Evolutionary Genomics Group, Departamento de Producción Vegetal y Microbiología, Universidad Miguel Hernández, San Juan de Alicante, 03550 Alicante, Spain

[2]Institute of Hydrobiology, Department of Aquatic Microbial Ecology, Biology Center of the Academy of Sciences of the Czech Republic, České Budějovice, Czech Republic

[3]Cavanilles Institute of Biodiversity and Evolutionary Biology, University of Valencia, Burjassot, E-46100 Valencia, Spain

*Correspondence: Francisco Rodriguez-Valera, E-mail: frvalera@umh.es

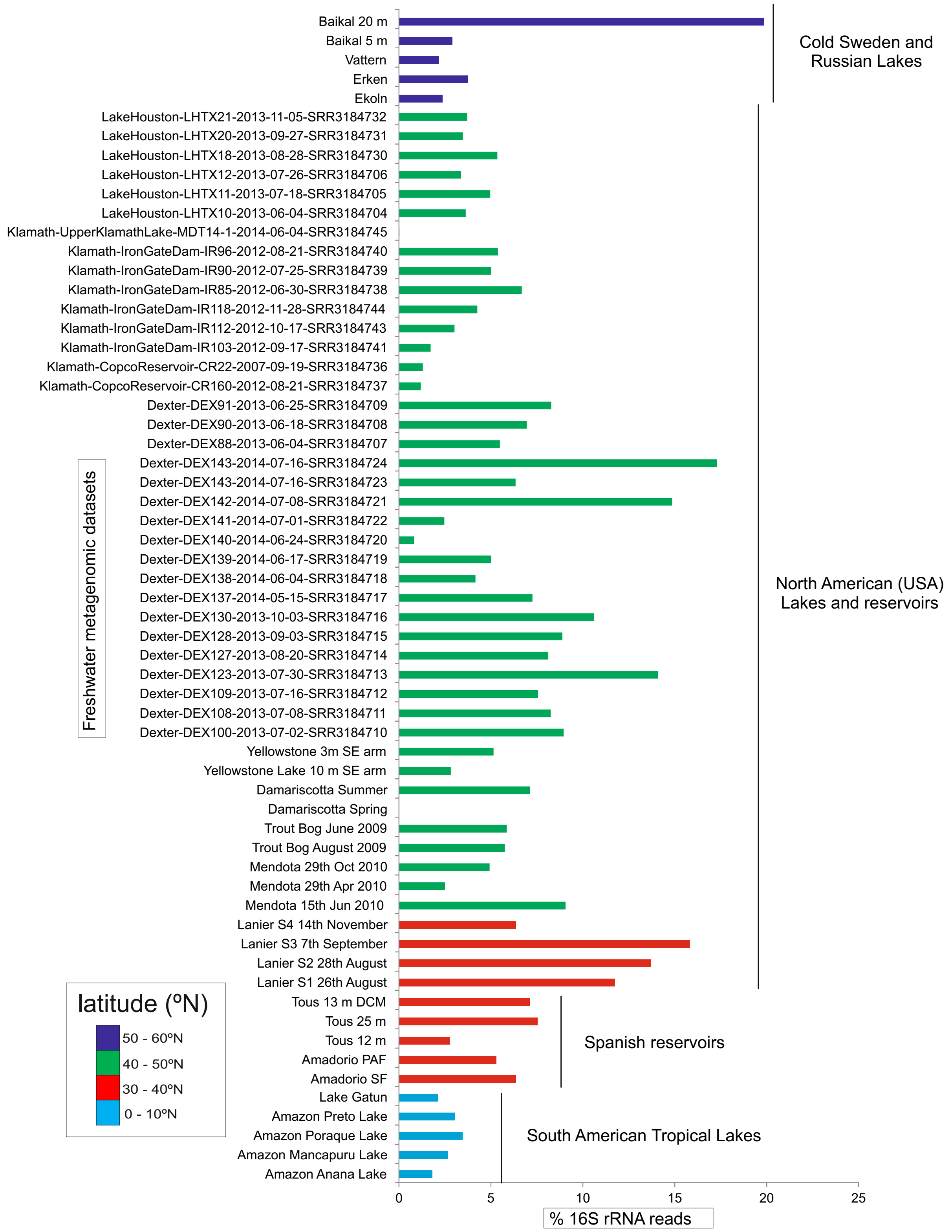

Supplementary Figure 1. % of 16S rRNA verrucomicrobial fragments along different freshwater metagenomics datasets. Latitudes of the different freshwater bodies shown are colour coded.

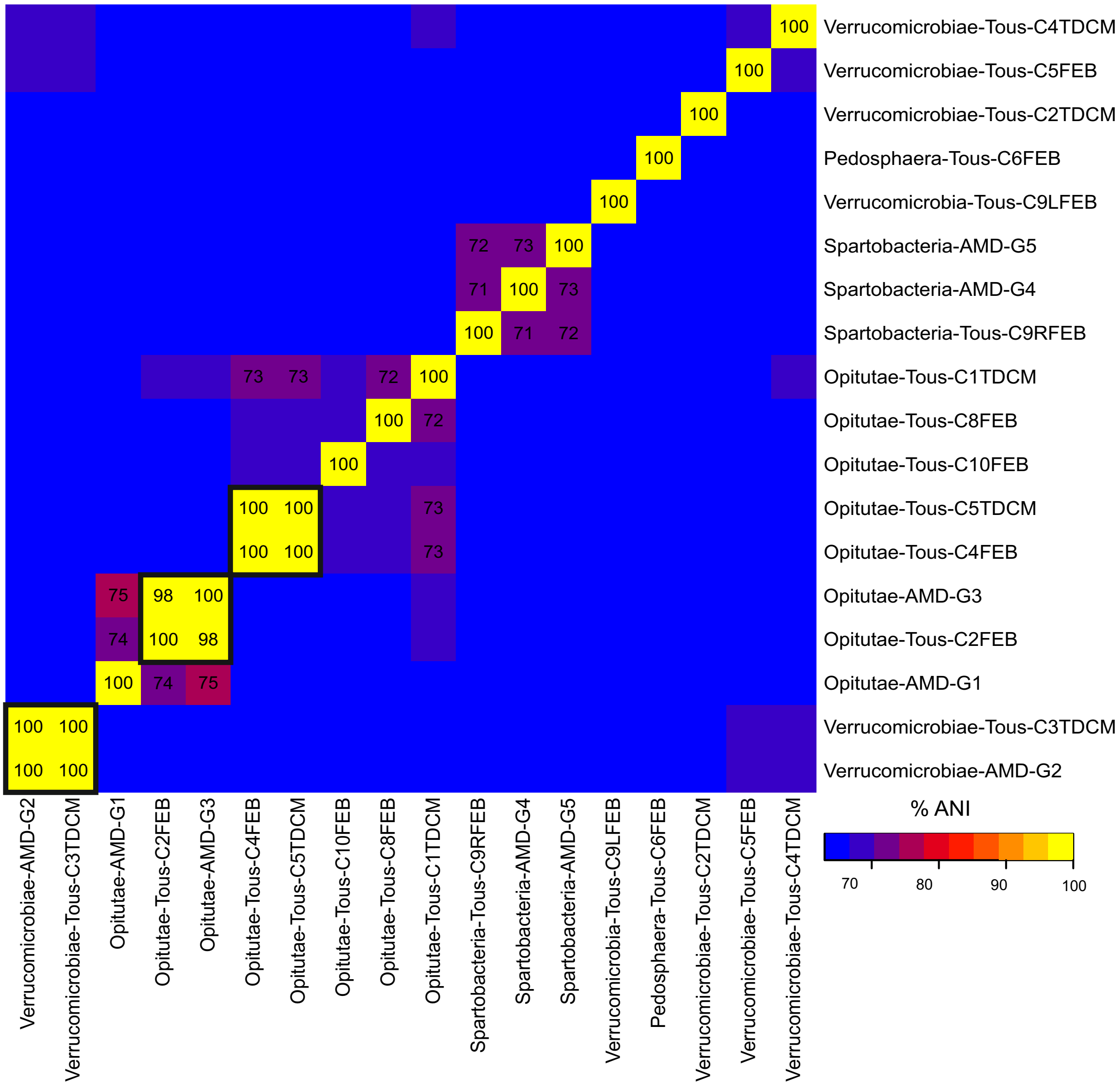

Supplementary Figure 2. Average nucleotide identity (ANI) among the reconstructed *Verrucomicrobia* genomes described in this work.

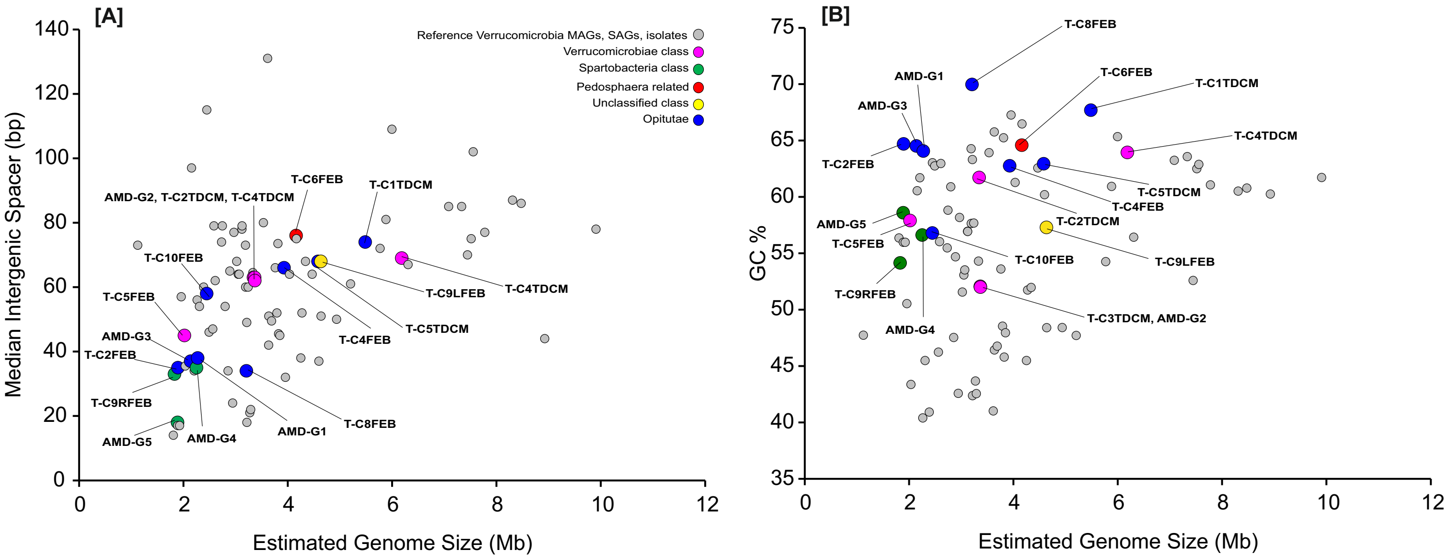

Supplementary Figure 3. *Verrucomicrobia* estimated genome size (Mb) versus median intergenic spacer (bp) and GC content scatter plots. **(A)** Estimated genome size (Mb) versus Median intergenic spacer (bp) **(B)** Estimated genome size (Mb) versus GC content of *Verrucomicrobia* MAGs, SAGs and isolates. Reference *Verrucomicrobia* are grey colour codified. Tous and Amadorio MAGs are differently colour codified according to their class affilitation.

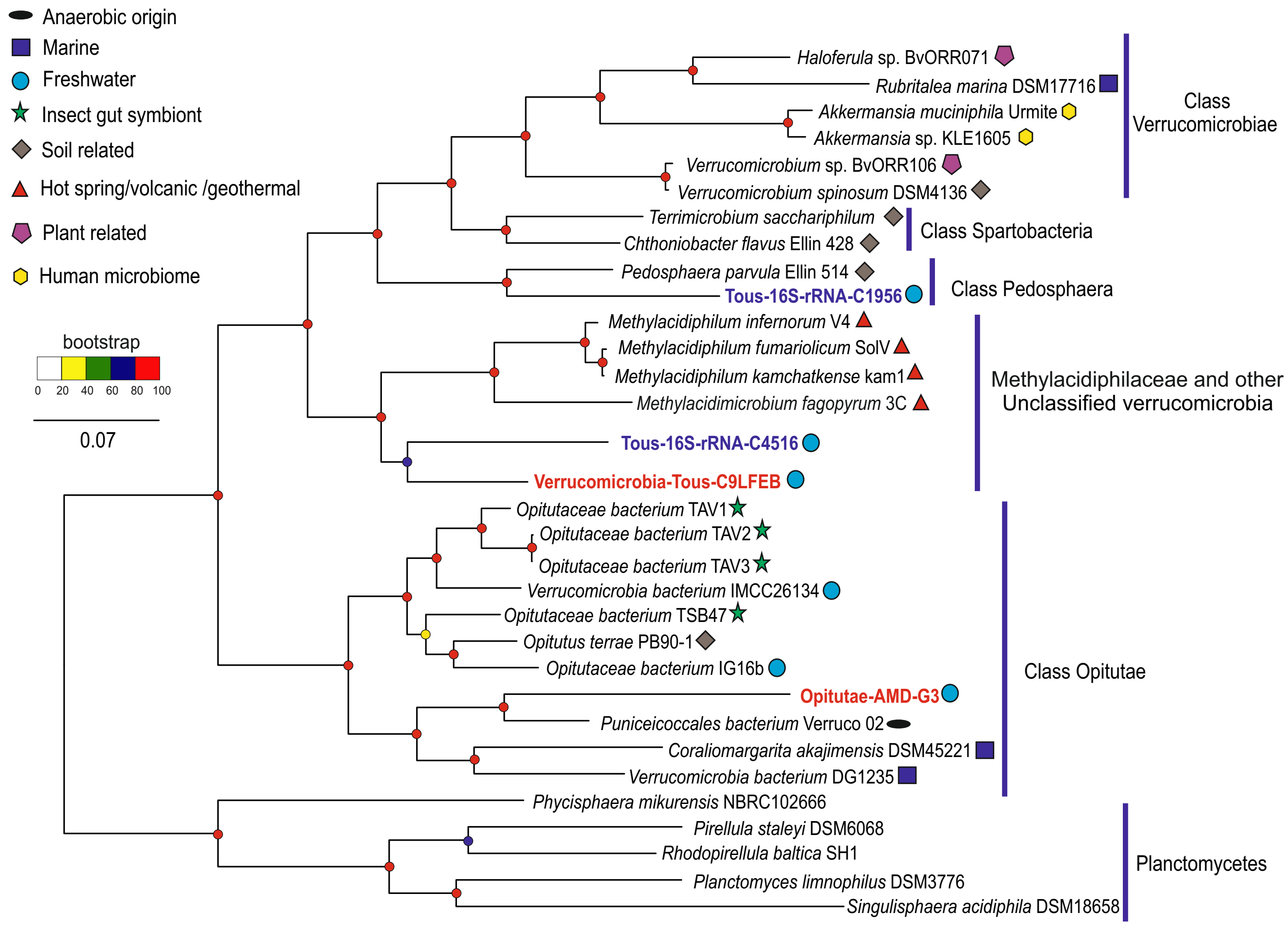

Supplementary Figure 4. *Verrucomicrobia* 16S rRNA phylogeny. Twenty three reference *Verrucomicrobia* and 5 *Planctomycetes* 16S rRNA sequences were used. 16S rRNA sequences belonging to MAGs from Tous and Amadorio are red coloured. Unbinned 16S rRNA sequences from Tous or Amadorio contigs >10 kb are blue coloured.

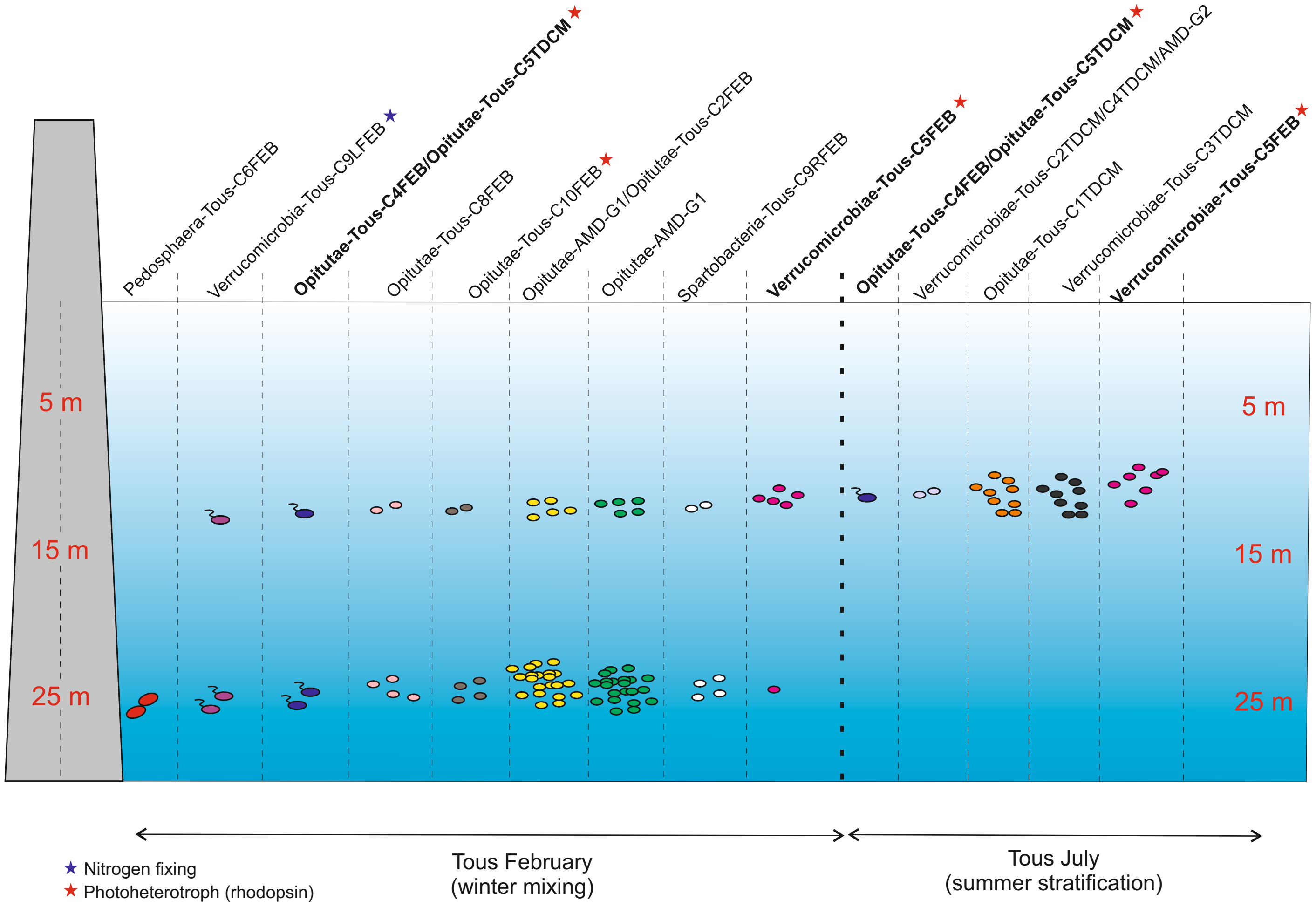

Supplementary Figure 5. Distribution pattern of *Verrucomicrobia* MAGs in Tous reservoir during winter (mixed water column samples of 12 and 25 m) and summer (stratified water column sample of 13 m) regimes. We estimated the distribution of each MAG based on the RPKG (reads per Kb of genome per Gb of metagenome) of Tous metagenomic datasets. MAGs containing rhodopsins (red star) or nitrogen fixing genes (blue star) are indicated. *Verrucomicrobia* MAGs which are present both in February and July samples are stated in bold.

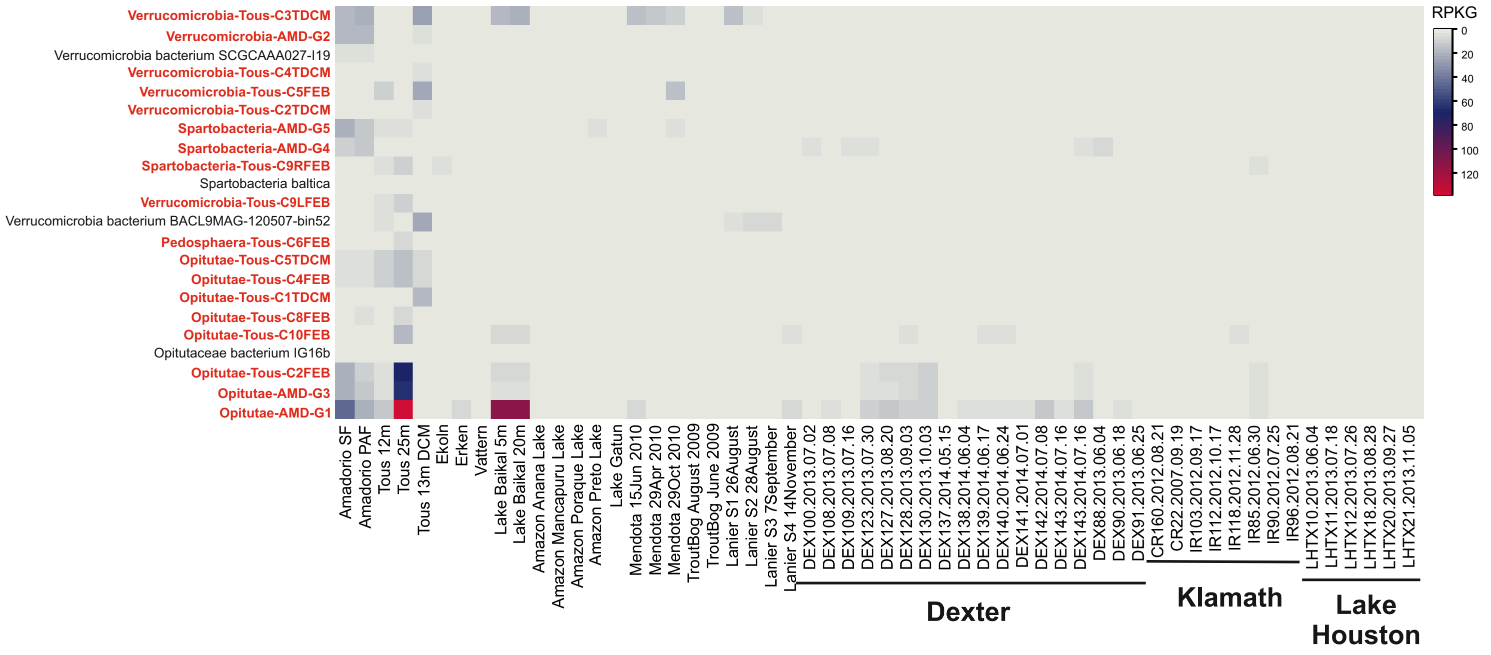

Supplementary Figure 6. Abundances of *Verrucomicrobia* genomes along different freshwater metagenomic datsets. Tous and Amadorio MAGs described in this study are red coloured in the Y axis. Reference freshwater, marine, brackish, soil and plant related *Verrucomicrobia* genomes (either isolates, MAGs or SAGs) are also shown in the Y axis. X axis shows the different freshwater metagenomic datasets used for the abundance estimation. Abundances are displayed with RPKG value (Reads per Kb of genome per Gb of metagenome).

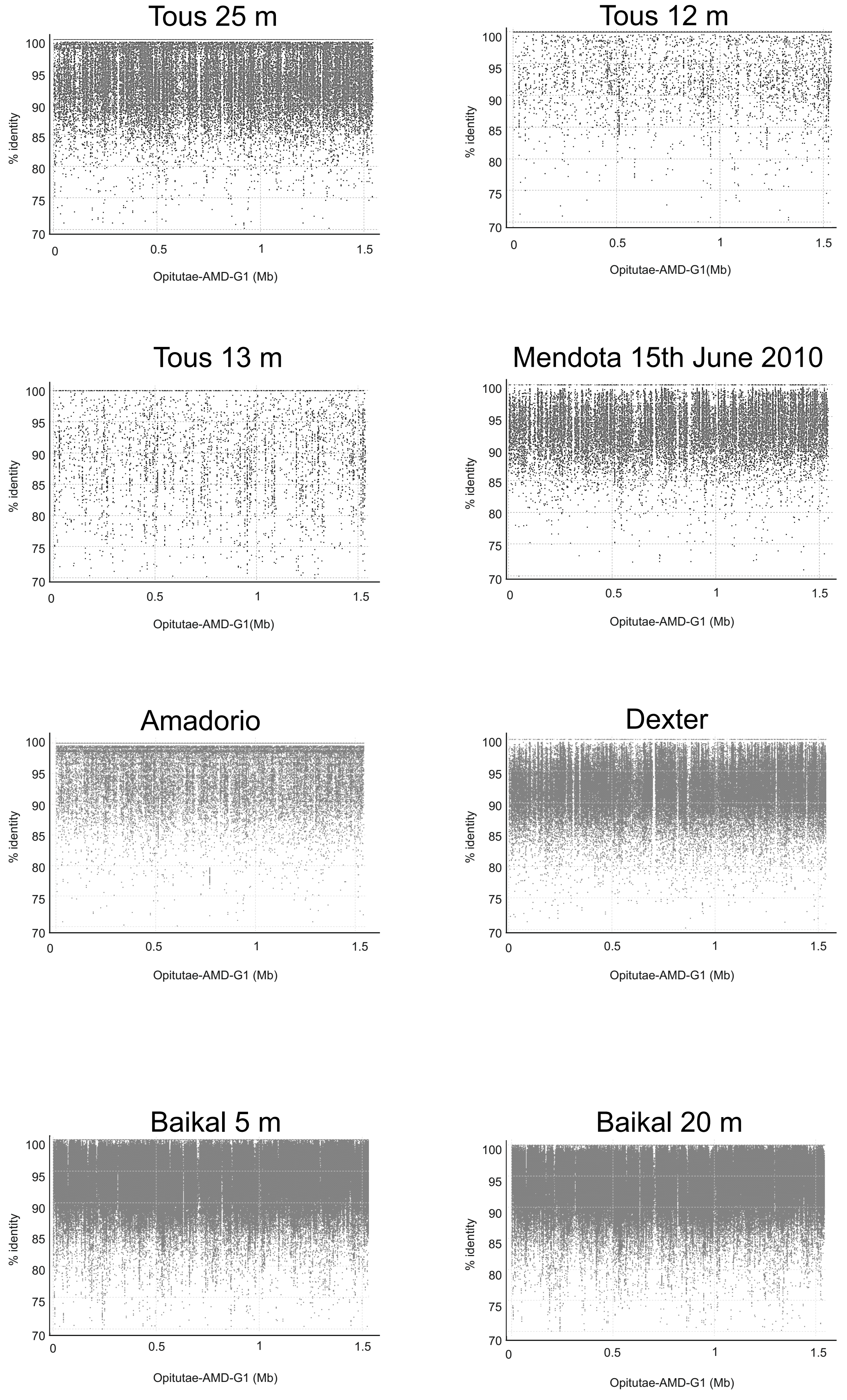

Supplementary Figure 7. Opitutae-AMD-G1 recruitment plots for different freshwater metagenomic datasets. X axis represents position in the genome (Mb). Y axis represents the % of identity of each metagenomic read mapped to the genome. A minimum of 70 % of identity and 50 bp of alignment lengths were used as threshold. Datasets used: Amadorio (Ghai *et al* 2014) and Tous reservoir (Cabello-Yeves *et al* 2017), Dexter reservoir (SRR3184716), Lake Baikal (PRJNA396997, SRR5896114 and SRR5896115) and Lake Mendota June 2010 (SRR408160).

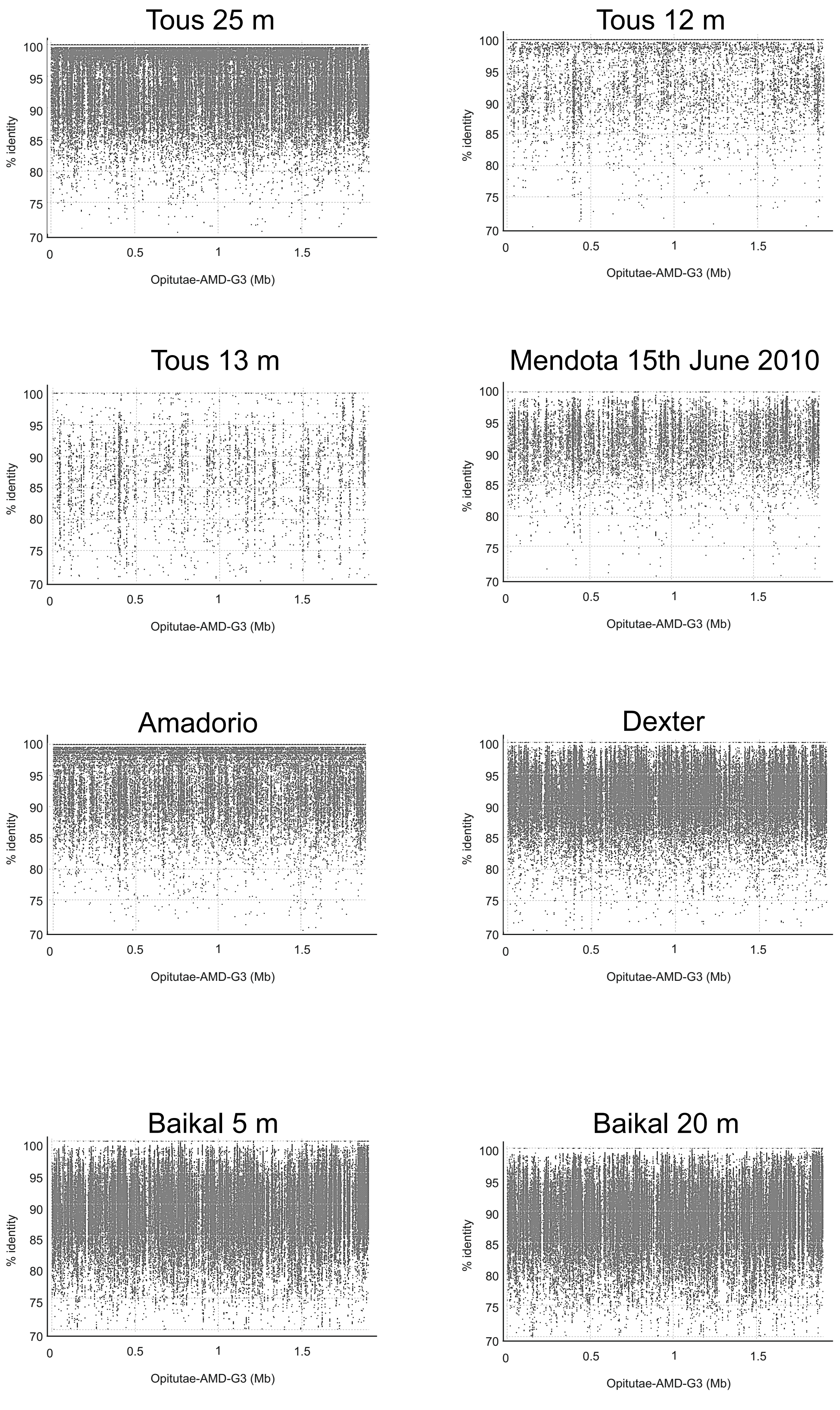

Supplementary Figure 8. Opitutae-AMD-G3 recruitment plots for different freshwater metagenomic datasets. X axis represents position in the genome (Mb). Y axis represents the % of identity of each metagenomic read mapped to the genome. A minimum of 70 % of identity and 50 bp of alignment lengths were used as threshold. Datasets used: Amadorio (Ghai *et al* 2014) and Tous reservoir (Cabello-Yeves *et al* 2017), Dexter reservoir (SRR3184716), Lake Baikal (PRJNA396997, SRR5896114 and SRR5896115) and Lake Mendota June 2010 (SRR408160).

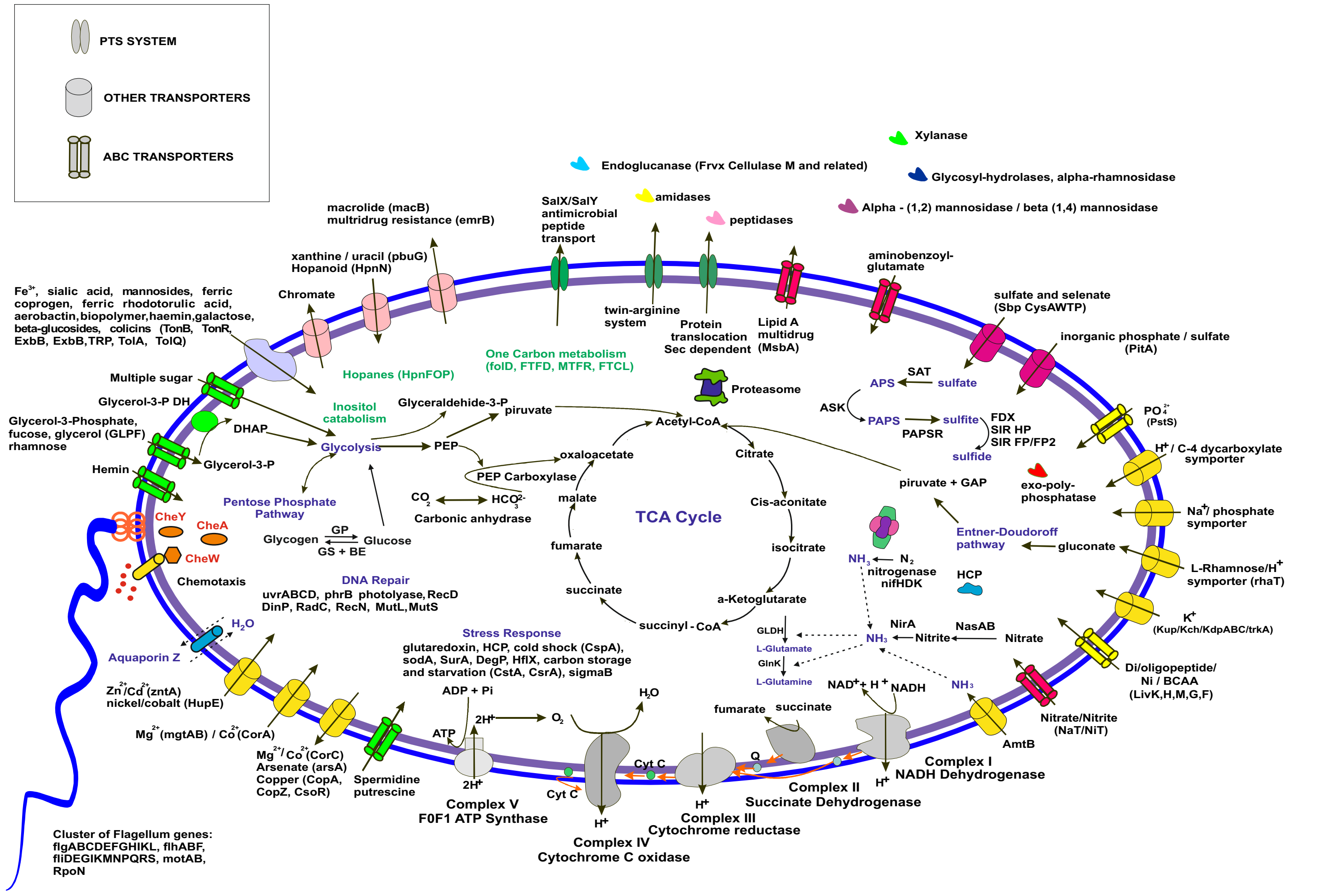

Supplementary Figure 9. Metabolic overview of the unclassified genome Verrucomicrobia-Tous-C9LFEB. All pathways shown are complete in the genome.

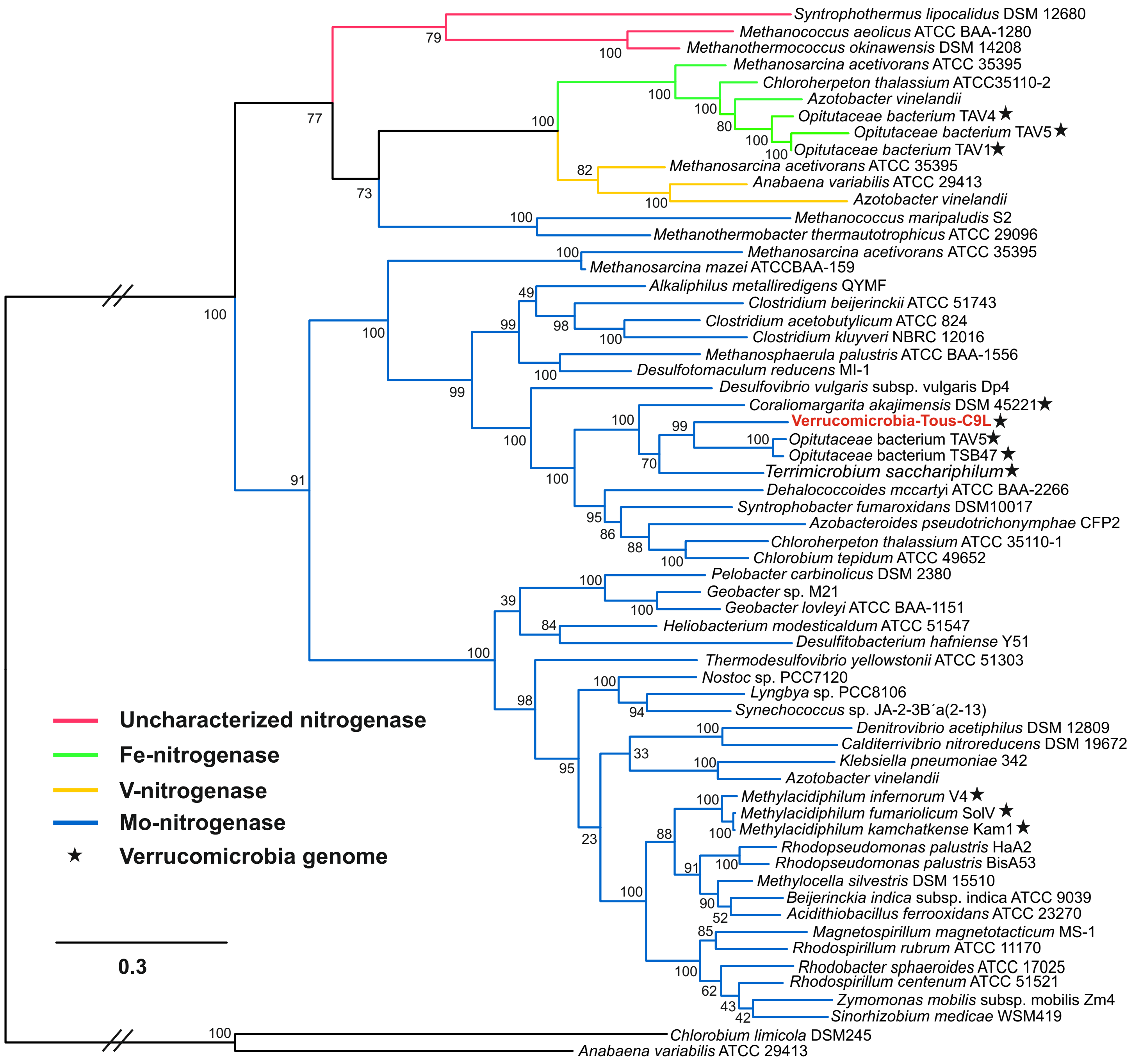

Supplementary Figure 10. Phylogeny of the nifHDK protein concatamer. Different molybdenum, vanadium and iron nitrogenases from different bacteria are represented as previously described (Boyd, 2000; Boyd *et al.*, 2011; Boyd and Peters, 2013; McGlynn *et al.*, 2013; Boyd *et al.*, 2015). Concatenations of paralogous proteins involved in the synthesis of chlorophyll/bacteriochlorophyll (Bch/ChlLNB) were used to root the phylogeny. Verrucomicronia representatives are star-symbolized. The novel nitrogenase from the freshwater Verrucomicrobia-Tous-C9LFEB is red coloured.

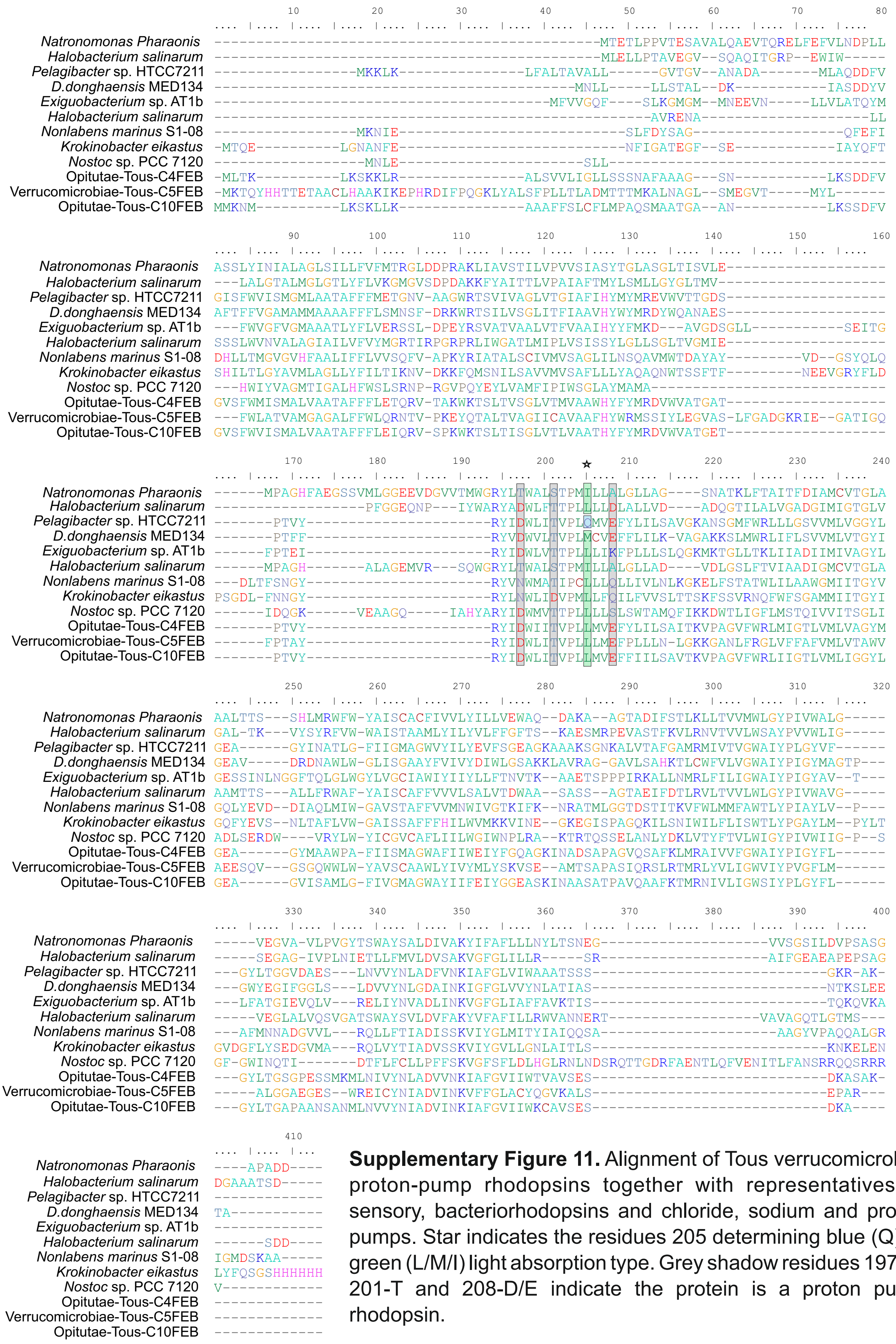

Supplementary Figure 11. Alignment of Tous verrucomicrobial proton-pump rhodopsins together with representatives of sensory, bacteriorhodopsins and chloride, sodium and proton pumps. Star indicates the residues 205 determining blue (Q) or green (L/M/I) light absorption type. Grey shadow residues 197-D, 201-T and 208-D/E indicate the protein is a proton pump rhodopsin.
